# Dissecting the Function of Hippocampal Oscillations in a Human Anxiety Model

**DOI:** 10.1523/JNEUROSCI.1834-16.2017

**Published:** 2017-07-19

**Authors:** Saurabh Khemka, Gareth Barnes, Raymond J. Dolan, Dominik R. Bach

**Affiliations:** ^1^Division of Clinical Psychiatry Research, University of Zurich, 8032 Zürich, Switzerland,; ^2^Neuroscience Centre Zurich, University of Zurich, 8006 Zürich, Switzerland,; ^3^Wellcome Trust Centre for Neuroimaging, University College London, London WC1N 3BG, United Kingdom,; ^4^Max Planck UCL Centre for Computational Psychiatry and Ageing Research, University College London, London WC1N 3BG, United Kingdom, and; ^5^Department of Psychiatry, Psychotherapy, and Psychosomatics, University of Zurich, 8032 Zurich

**Keywords:** anxiety, approach–avoidance, cingulate cortex, hippocampus, MEG, theta oscillations

## Abstract

Neural oscillations in hippocampus and medial prefrontal cortex (mPFC) are a hallmark of rodent anxiety models that build on conflict between approach and avoidance. Yet, the function of these oscillations, and their expression in humans, remain elusive. Here, we used magnetoencephalography (MEG) to investigate neural oscillations in a task that simulated approach–avoidance conflict, wherein 23 male and female human participants collected monetary tokens under a threat of virtual predation. Probability of threat was signaled by color and learned beforehand by direct experience. Magnitude of threat corresponded to a possible monetary loss, signaled as a quantity. We focused our analyses on an a priori defined region-of-interest, the bilateral hippocampus. Oscillatory power under conflict was linearly predicted by threat probability in a location consistent with right mid-hippocampus. This pattern was specific to the hippocampus, most pronounced in the gamma band, and not explained by spatial movement or anxiety-like behavior. Gamma power was modulated by slower theta rhythms, and this theta modulation increased with threat probability. Furthermore, theta oscillations in the same location showed greater synchrony with mPFC theta with increased threat probability. Strikingly, these findings were not seen in relation to an increase in threat magnitude, which was explicitly signaled as a quantity and induced similar behavioral responses as learned threat probability. Thus, our findings suggest that the expression of hippocampal and mPFC oscillatory activity in the context of anxiety is specifically linked to threat memory. These findings resonate with neurocomputational accounts of the role played by hippocampal oscillations in memory.

**SIGNIFICANCE STATEMENT** We use a biologically relevant approach–avoidance conflict test in humans while recording neural oscillations with magnetoencephalography to investigate the expression and function of hippocampal oscillations in human anxiety. Extending nonhuman studies, we can assign a possible function to hippocampal oscillations in this task, namely threat memory communication. This blends into recent attempts to elucidate the role of brain synchronization in defensive responses to threat.

## Introduction

Anxiety comprises a suite of behaviors to account for potential threat, enabling an organism to strike a normatively optimal balance in the face of competing goals (Bach, 2015, 2017). Using rodent approach–avoidance conflict tests, such as the elevated plus maze (EPM) or open-field test (OFT), a plethora of lesion and drug infusion studies have implicated the ventral hippocampus and medial prefrontal cortex (mPFC) in the control of such behaviors (Gray and McNaughton, 2000; Kjelstrup et al., 2002; Trent and Menard, 2010; Weeden et al., 2015; Ito and Lee, 2016). In line with these findings, a recent lesion study suggested a similar role of the human homolog, the anterior hippocampus, in anxiety-like behavior (Bach et al., 2014). In rodent anxiety tests, increased ventral hippocampal theta synchronization with mPFC, and increased theta power in hippocampus, is observed when comparing these situations to a familiar environment (Adhikari et al., 2010; Padilla-Coreano et al., 2016). However, the function of these oscillations and their expression in humans is currently unclear. In this proof-of-principle study, we used an operant conflict test to demonstrate hippocampal power increase in human anxiety and hippocampal synchronization with mPFC and to investigate different possible causes.

In rodents, hippocampal and mPFC theta oscillations have been suggested to signal aversive or safe aspects of anxiety situations (Adhikari et al., 2010; Padilla-Coreano et al., 2016). However, innate anxiety tests, like the EPM or OFT, involve multiple possible threat features, which may be learned in plastic circuits or hard-wired. This precludes better characterizing the function of theta oscillations in these tests. On the other hand, during fear conditioning, a more controlled situation without goal conflict, theta and gamma synchronization between amygdala, hippocampus, and PFC has been implicated in the communication of threat memory (Stujenske et al., 2014). Here, we speculated that hippocampal oscillations, and anxiety-related synchronization, may preferentially relate to learned threat probability, but not to other aversive features, such as explicitly signaled magnitude of threat.

**Figure 1.**
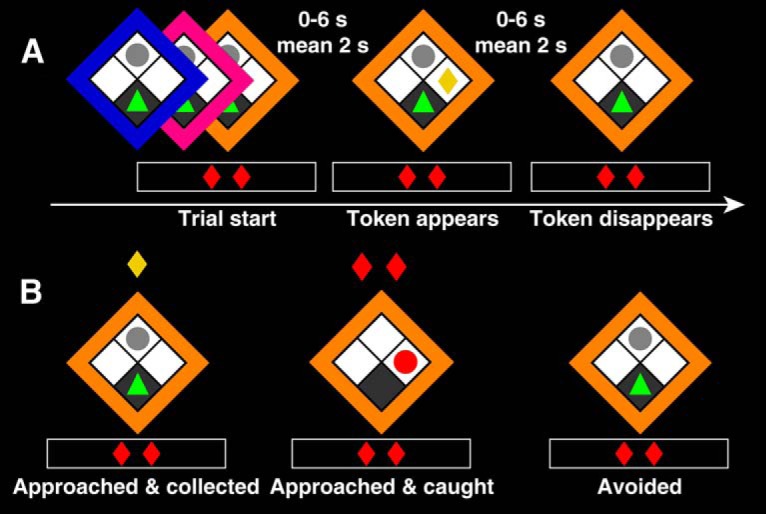
Virtual computer game. ***A***, At trial start, the player (green triangle) is placed into a “safe place” on a 2 × 2 grid with one of three different frame colors, representing threat level of a sleeping predator (gray circle). Red tokens signal potential token loss upon being caught (0–5). After a random interval, a monetary token (yellow diamond) appears. If not collected, it disappears after a random interval. ***B***, Possible outcomes depend on participants' choice and chance.

To this end, we capitalized on a previously established human approach–avoidance conflict model of anxiety (Bach et al., 2014; Korn et al., 2017), embedded in a virtual computer game (Bach, 2015, 2017), while recording magnetoencephalography (MEG) to assess neural oscillations. On each trial of the game, a human player could collect a single monetary token under threat of getting caught by a virtual “predator”. Catch probabilities for three distinctly colored predators were learned by experience beforehand (termed “threat level”). Being caught incurred a monetary loss that was explicitly signaled as a quantity on each trial (termed “potential loss”). At trial start, the player was presented with the predator color and the potential loss. After a random interval, the token appeared to create behavioral conflict (Fig. 1). We analyzed neural oscillations separately at both time points.

## Materials and Methods

### 

#### 

##### Datasets.

From the student and general population, 20 right-handed healthy participants (mean age ± SD, 24.3 ± 3.91 years; 10 female) were recruited in Zurich for a behavioral experiment (Experiment 1), and 25 right-handed healthy participants (22.9 ± 3.68 years; 14 female) took part in a MEG experiment in London (Experiment 2). All participants were fluent speakers of German or English, respectively, and had normal or corrected-to-normal vision. Two MEG participants were excluded from the final analysis: one did not complete the experiment and the other made large head movements (>0.5 cm) impairing source reconstruction.

The study protocol was in full accordance with the Declaration of Helsinki. All participants gave written informed consent after being fully informed about the purpose of the study. The study protocol, participant information, and form of consent, were approved by research ethics committees (Kantonale Ethikkommission Zurich, University College London Research Ethics Committee).

##### Experimental task.

Participants performed an approach–avoidance conflict task embedded in a computer game (Fig. 1), modified from a previous study (Bach, 2015). Notably, this task involves only financial gains and losses, but previous work indicates that participants' behavior, in particular the relation of approach latency with expected loss, is not explained by economic theory and fits accounts of anxiety-like behavior derived from nonhuman anxiety tasks (Bach, 2015). To make the game usable for MEG, we segregated individual token presentations into separate trials.

On each trial, participants could collect one monetary token (approach motivation) under threat of getting caught by a predator and consequently losing an explicitly signaled number of tokens (avoidance motivation). Specifically, at the start of each trial (Fig. 1*A*), the human player was in a “safe place”, the bottom grid block in a 2 × 2 diamond grid, and was tasked to decide whether or not to collect a token that would come up in the left or the right grid block. The predator was “sleeping” opposite the safe place, and could become active in a homogenous Poisson process when the human player was outside the safe place, in which case it would catch the player. Three frame colors (blue, pink, or orange) represented the threat levels, i.e., the Poisson wake-up rate of the predators. Wake-up rates were set to result in a catch probability of 0.1, 0.2, or 0.3, for the three predators, if the player was outside of the safe place for 100 ms, a value established in previous work (Bach, 2015). Threat probabilities were learned by experience beforehand in 36 training trials with zero token loss, which did not count toward the performance-based remuneration. Crucially, threat probabilities were not explicitly instructed. Below the grid, potential loss on the current trial was indicated by red diamonds and varied between 0 and 5.

After a variable time interval, randomly drawn from a gamma distribution with parameters *k* = 2, θ = 1, and mean of 2 s, truncated at 6 s, the token appeared. In case the player did not collect the token, it disappeared after a variable time, drawn from the same distribution, and the trial continued for another 1 s. If the token was collected (Fig. 1*B*), the trial continued until the same predetermined end time. If the player got caught, it disappeared, the predator turned red and stayed on the screen until the predetermined end time. The next trial started after a random intertrial interval (ITI) drawn from the same distribution truncated at 4 s, during which the screen was blank. Participants were presented with 648 trials in Experiment 1 and 540 trials in Experiment 2, evenly distributed across six different token losses and three different threat levels in pseudorandom order.

Participant's payment depended on performance in six trials randomly drawn after the experiment and excluding the 36 training trials. The experiment was programmed in Cogent (Version 2000v1.25; www.vislab.ucl.ac.uk/Cogent) under MATLAB 7.14 (MathWorks).

##### MEG data acquisition.

MEG signals were recorded in a magnetically shielded room with a 275-channel Canadian Thin Film system with superconducting quantum interface device (SQUID)-based axial gradiometers, a hardware anti-alias filter of 150 Hz cutoff frequency, and digitization rate of 600 Hz. Head positioning coils were attached to nasion, left, and right auricular sites, to provide anatomical coregistration, and allowed continuous head localization. Synchronizing markers were written into the MEG data file for precise detection of trial start, token appearance, and trial end. A projector displayed the computer game on a screen (∼0.8 m distance from the participant). Participants made responses with a button box, and eye blinks were monitored using an eye tracker.

This type of MEG system has been successfully used in the past across different laboratories to demonstrate hippocampal oscillations. This includes theta oscillations during navigation, well known from nonhuman electrophysiology (Cornwell et al., 2012; Kaplan et al., 2012), theta oscillations during memory recall, known from fMRI and animal electrophysiology (Guitart-Masip et al., 2013), hippocampal-mPFC phase coupling during decision making (Guitart-Masip et al., 2013), increased theta oscillations during memory encoding, a phenomenon well known from nonhuman electrophysiology (Backus et al., 2016), and theta-gamma coupling during replay, another phenomenon from nonhuman electrophysiology (Poch et al., 2011). Furthermore, the approach has been used to replicate an fMRI experiment on stimulus novelty, showing increased hippocampal theta oscillations with novelty (Garrido et al., 2015). Simultaneous intracranial EEG and MEG recordings have also provided support for the validity of hippocampal source reconstruction in the gamma band (Dalal et al., 2013). In sum, the gradiometer system appears well suited to record oscillations from hippocampal sources. In terms of theoretic considerations, while there is greater attenuation of distant sources for gradiometers than for magnetometers, this is generally compensated for by an increased SNR due to better noise rejection performance. Under an assumption that the hippocampus is 8 cm away from the nearest sensor, then a 5 cm baseline gradiometer will provide 60% of the signal compared with a magnetometer. However, at the same time, the gradiometer offers typically a 100-fold improvement in far-field external noise rejection compared with the magnetometer.

#### Data analysis

##### Behavioral data analysis.

Statistical analysis of behavioral data were performed in R (www.r-project.org, v3.1.2). Because the data were unbalanced by design, we used linear mixed-effects models (lme4 package), which provide meaningful parameter estimates in this case, using a previously described method (Bach, 2015). All models had the following form:

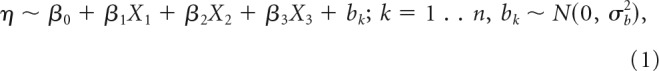
 where β_0_ is the group intercept, β_1…3_ are the fixed effects parameter vectors for three threat levels, six potential losses, and their interaction, and *b*_k_ is the random subject intercept. The linear predictor η is related to the data *y* through the identity link function for the approach latency data:

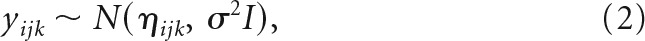
 and through the logit link function for binary choice data (i.e., approach–avoid):

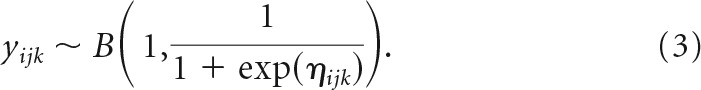
 This is equivalent to the *R* model formula:

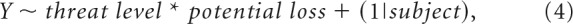
 where Y is the binary choice, or the approach latency. Fixed-effects F-statistics were computed using the R function anova. *P-*values were calculated using a conservative lower bound on the effective denominator degrees of freedom as

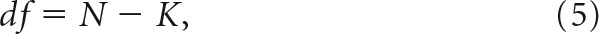
 where *N* is the number of observations and *K* is the number of all modeled fixed and random effects. Because the data are unbalanced, i.e., some participants made no approach responses for higher potential loss or threat level, the averaged approach latencies at higher potential loss or threat level will be biased by participants who are more likely to approach. This is why we estimated the approach latency from the model for illustration (lsmeans package). This approach takes the unbalanced dataset into account and estimates the mean approach latency that would be expected in a balanced dataset.

##### MEG data preprocessing.

MEG data analysis was conducted in SPM12 (Statistical Parametric Mapping, Wellcome Trust Centre for Neuroimaging, London, UK; http://www.fil.ion.ucl.ac.uk/spm/). Continuous data from each session were high-pass filtered at 0.1 Hz and low-pass filtered at 150 Hz using a fifth-order Butterworth filter, down-sampled to 150 Hz, and notch filtered at 50 and 100 Hz to remove mains noise. Data were down sampled to 300 Hz resolution. Epochs from 0 to 1000 ms relative to trial start and to token appearance of each trial were extracted separately. Epochs in which the interval between trial start and token appearance, or the interval between token appearance and trial end, were shorter than 1000 ms were discarded from further analysis. This excluded ∼26% of the trials as expected from the cumulative density function of the gamma distribution.

##### Source localization.

The linearly constrained minimum variance scalar beamformer spatial filter algorithm (implemented in DAiSS toolbox, https://github.com/SPM/DAiSS) was used to generate maps of source activity on a 5 mm grid. Coregistration to the Montreal Neurological Institute (MNI) brain template was based on three fiducial points: nasion, left, and right preauricular points. We used a single-shell head model to fit the inner skull surface of the inverse normalized SPM template to more precisely characterize the MEG forward model. The beamformer source reconstruction calculates a set of weights that maps the sensor data to time-series at the source locations. Our broad-band beamforming spatial filters were based on covariance matrix of all trials, in a frequency range of 1–150 Hz and a time window of 0–1000 ms relative to trial start or token appearance.

For each participant, we then created four normalized 3D source power images depicting the following contrasts: difference between high threat and low threat level, linear effect of potential losses across different threat levels, quadratic effect of potential losses across different threat levels, and interaction between threat levels and potential losses. The resulting images were smoothed using a Gaussian kernel of 10 mm FWHM (Guitart-Masip et al., 2013). We then performed a second level one-sample *t* test on smoothed contrast images from all the participants (df = 22). All statistical parametric maps were thresholded at *p* < 0.001 uncorrected, and small volume corrected for family wise error at *p* < 0.05 using Gaussian random-field theory at the cluster level (Worsley et al., 1996) within the bilateral hippocampus defined by the AAL toolbox (Tzourio-Mazoyer et al., 2002).

##### Lateralization.

To assess the laterality of our main finding, we extracted averaged power from the significant clusters and contralaterally mirrored hippocampal regions (i.e., the clusters flipped about the midline). Because this analysis is biased toward exposing a difference, we also extracted data from left and right hippocampus separately. These data were analyzed in a 3 (threat level) × 2 (hemisphere) ANOVA.

##### Controlling for behavioral variables.

To exclude that behavior (decision to approach or approach latency) explained our findings, we extracted averaged power from both clusters on a trial-by-trial basis. Because behavior is strongly coupled to threat level and potential loss, the number of instances for each combination of experimental condition and behavioral response is extremely unbalanced. This is why we departed from our previous ANOVA approach and analyzed these data in a full hierarchical linear mixed-effects model in line with behavioral data analysis, using the *R* formula:

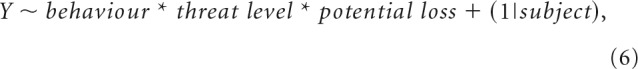
 where behavior corresponds to the approach–avoidance decision (control analysis 1) or to the approach latency (control analysis 2, accounting only for data on trials where participants chose to approach).

##### Comparing threat level and potential loss.

To compare the effect of threat level and token loss in an unbiased region-of-interest, we extracted theta power for all trials from all image voxels within the bilateral hippocampus. For each individual voxel and for the average across all voxels, we compared a reduced model containing either the linear effect of threat level together with subject intercepts, or the linear effect of token loss together with subject intercepts. For both models, we computed Akaike information criterion (AIC). An absolute AIC difference of >3 was regarded as decisive (Penny et al., 2004).

##### Decomposition into frequency bands.

We extracted power from the significant clusters separately for five frequency bands: theta (1–8 Hz), alpha (8–12.5 Hz), beta (12.5–30 Hz), gamma (30–80 Hz), and high gamma (80–150 Hz). To define the frequency range of theta oscillations in humans, we drew on previous work exploring their distinctive association with gamma oscillations at species-specific frequencies. In rodents, these appear to occur between 4 and 12 Hz (Adhikari et al., 2010). In contrast, intracranial recordings have revealed that human hippocampal theta oscillations occur in an overall lower frequency range (1–8 Hz) (Jacobs, 2014). Definition of the other frequency bands was based on conventions in the field.

##### Time-frequency decomposition.

To analyze the evolution of theta activity at different time points and frequencies, we extracted all sources from the significant cluster and obtained time-frequency decomposition using Morlet wavelets. We computed mean power per subject and condition for each time point (0–1000 ms at 3 ms resolution) and frequency (1–150 Hz at 1 Hz resolution). These were then statistically analyzed by computing a two-tailed *t* test comparing high and low threat level for each data point. To account for multiple comparisons across time points or across frequencies, results were cluster-level corrected using a random permutation test on the trial labels (Maris and Oostenveld, 2007).

##### Modulation of gamma power envelope.

Gamma power modulation at theta frequency may indicate theta phase–gamma power coupling. To address gamma power modulation, we first averaged power at each time point across the predefined gamma band (30–80 Hz) and thus produced a time series of the gamma power envelope for each trial. For this power envelope, we obtained time-frequency decomposition using Morlet wavelets. We discarded all frequencies >30 Hz as they have limited interpretability. These data were averaged over time points and trials for each condition. We then averaged either across all conditions (overall gamma envelope), or computed the difference between high and low threat. These data were averaged within theta, alpha, and beta frequency band, and analyzed in a univariate (frequency band) ANOVA or threat level × frequency band ANOVA. To address evoked (time-locked) modulation of the gamma envelope, we first averaged the gamma envelope within conditions and then repeated this analysis.

##### Synchronization analysis.

To estimate synchronization of hippocampal theta oscillations with the rest of the brain, we computed the phase lag index (PLI; Stam et al., 2007). We extracted trial-by-trialwise time series for the time window following token appearance, for the first principal component of all sources within the significant cluster (seed source), and then for all other sources in the brain. These time series were filtered (1–8 Hz bidirectional fourth-order Butterworth) and Hilbert transformed to compute instantaneous phase φ(*t*, *n*) at time *t* for source *n*. Phase lag index was then calculated for each trial as follows:

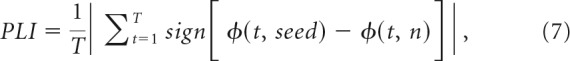
 where φ(*t*, *seed*) represents instantaneous phase of source in the significant cluster at time *t*. PLI will range from 0 to 1, where a PLI of 0 indicates no coupling or randomly distributed phase angles and a PLI of 1 indicates constantly positive or negative phase angle across time points and thus tight coupling between two sources-of-interest. The PLI measure is less prone than other synchronization measures to the influences of volume conduction from a strong source (Stam et al., 2007; Kaplan et al., 2014). PLI between seed source and any other source in the brain was averaged across trials for each condition, written onto the 5 mm resolution source grid, averaged within each condition, and smoothed with a 10 mm FWMH Gaussian kernel before entering them into a second level statistical analysis. Statistical parametric maps were thresholded at *p* < 0.001 uncorrected, and small volume corrected for familywise error at *p* < 0.05 using Gaussian random field theory at the cluster level (Worsley et al., 1996) within an anatomical mPFC mask defined by combining BA 8-11, 44-47 in the AAL toolbox (Tzourio-Mazoyer et al., 2002), and restricting this mask to the medial cortex surface (±4 mm about the midline).

To make plausible that these results are not biased by condition differences in difference phase angle, we extracted for each trial and time point the difference phase angle between seed source and all sources within the significant mPFC cluster from the PLI analysis. This showed no large overall phase angle differences, thus rendering the analysis of PLI unproblematic.

## Results

### 

#### Increasing threat level, or potential loss, enhances passive avoidance and behavioral inhibition

**Figure 2.**
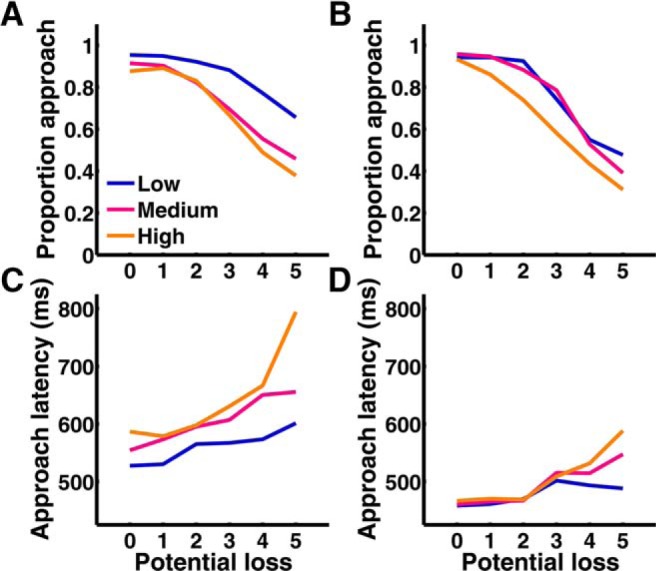
Behavioral results. Proportion of approach responses (***A***, ***B***) and approach latency (***C***, ***D***) for a behavioral experiment (*n* = 20; ***A***, ***C***) and the MEG experiment (*n* = 23; ***B***, ***D***). Approach latency is estimated from a linear mixed-effects model to account for the unbalanced data structure.

Figure 2 shows that participants adapted their behavior across varying level of threat and potential loss in a behavioral control sample, and in the MEG experiment. The proportion of approach responses significantly decreased with increasing threat level, and with potential loss (Fig. 2*A*,*B*; Table 1), similar to passive avoidance observed in rodents during anxiety tests. Also, when participants made an approach response, approach latency was longer at high threat level or potential loss (Fig. 2*C*,*D*; Table 1). This suggests behavioral inhibition relates to expected loss. These results replicate previous reports with a similar operant conflict game in which participants collected tokens cumulatively (Bach, 2015, 2017), whereas here potential loss did not depend on previous actions. We also noted that participants' behavior separated between high- and low-threat situations, but less so between medium and high threat level. A behavioral effect of varying threat level and potential loss on approach latency indicates that our model captures approach–avoidance conflict in humans, and hints at the cross-species comparability of the model.

**Table 1.** Effect of threat level, potential loss, and their interaction on proportion of approach responses, and approach latency in control and MEG study, as estimated in a linear mixed effects model on single trial data

	*F*	*p*	df
Effect on approach responses			
Behavioral experiment 1 (*n* = 20)			
Threat level	109.14	<0.001	2; 12,276
Potential loss	346.78	<0.001	5; 12,276
Threat level × potential loss	1.67	0.08	10; 12,276
MEG experiment 2 (*n* = 23)			
Threat level	27.85	<0.001	2; 12,380
Potential loss	438.78	<0.001	5; 12,380
Threat level × potential loss	7.18	<0.001	10; 12,380
Effect on approach latency			
Behavioral experiment 1 (*n* = 20)			
Threat level	29.42	<0.001	2; 9268
Potential loss	42.69	<0.001	5; 9268
Threat level × potential loss	4.65	<0.001	10; 9268
MEG experiment 2 (*n* = 23)			
Threat level	3.54	0.029	2; 9082
Potential loss	20.84	<0.001	5; 9082
Threat level × potential loss	2.29	0.019	10; 9082

#### Hippocampal oscillations relate to threat probability

**Figure 3.**
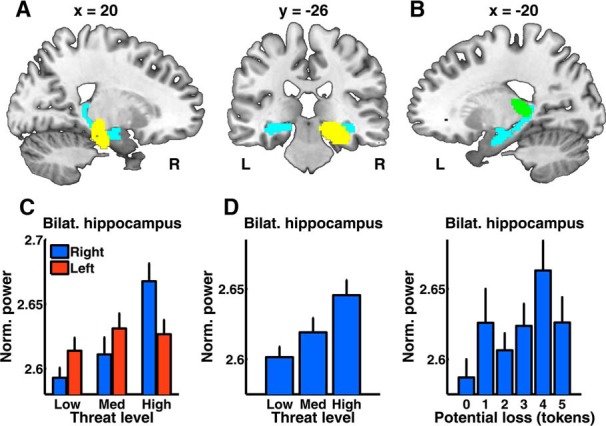
Estimated source power relates to threat level. ***A***, Right-hemispheric cluster for which estimated power increases with threat level (yellow) within an anatomically defined region-of-interest (bilateral hippocampus, light blue), visualized on a template brain image (*p* < 0.05 FWE). ***B***, Left-hemispheric cluster extending into the tip of the posterior hippocampus for which estimated power decreased with threat level (green). No voxel outside these two clusters showed such relationship at a voxel selection threshold of *p* < 0.001. ***C***, Mean normalized power in the hippocampus region-of-interest, for three different threat levels, shown as condition mean corrected for hemisphere mean across conditions, and SE of difference from participant/hemisphere mean. Power to threat level relation was more pronounced in right than left hemisphere. ***D***, Mean theta power averaged within the hippocampus region-of-interest for threat level and potential loss. Data are shown as condition mean and SE of difference from participant mean.

Upon token appearance, but not at trial start, we observed significantly greater power for high > low threat level in a cluster overlapping with the right mid-hippocampus (Table 2; Fig. 3*A*). We also observed a power decrease for high > low threat level in a cluster overlapping with the left posterior hippocampus and extending into the thalamus (Fig. 3*B*). We extracted power from each cluster and averaged across voxels. For both clusters, power at medium threat level was different from the high-threat level (one-tailed *t* tests, *p* < 0.05) but not from the low-threat level (*p* > 0.10), indicating a nonlinearity in the threat level–power relation. However, in a one-way ANOVA, the quadratic term for threat level was not significant (*p* > 0.10), indicating that this nonlinearity may be a chance variation. When comparing the significant clusters to a contralateral region (cluster mask flipped about the midline), we found a significantly greater influence of threat in one hemisphere than in the other. To avoid any bias induced by the cluster-defining contrast, we then extracted power from the anatomical region-of-interest and averaged separately for each hemisphere. Again, we observed a hemisphere × threat level interaction for both contrasts (mid-hippocampus: *F*_(1,44)_ = 4.04, *p* = 0.024; posterior hippocampus: *F*_(1,44)_ = 5.55, *p* = 0.007; Figure 3*C*). This suggests that the threat–power relationship is truly lateralized. Next, we expanded our field of view and analyzed power across the entire brain. No other brain region expressed a power relation with threat level, potential loss, or their interaction, even without correction for multiple comparisons.

**Table 2.** MEG findings

Hemisphere	*x*	*y*	*z*	Cluster size, mm^3^	Overlap with ROI mask, mm^3^	Peak *z* value
Token appearance: high threat > low threat						
Mid-hippocampus						
R	20	−24	−16	6256	1088	3.60
Posterior hippocampus/thalamus						
L	−12	−30	4	3504	464	4.18
Token appearance, PLI with respect to averaged sources in mid-hippocampus cluster: high threat > low threat						
Medial frontal gyrus (BA 10)						
Bilateral	−2	60	0	744		3.40

Results are cluster-level corrected for familywise error within the anatomically defined region-of-interest (bilateral hippocampus or bilateral mPFC) at *p* < 0.05 (cluster defining threshold of *p* < 0.001). Coordinates and peak *z* values refer to overall peak of the unmasked cluster.

#### Hippocampal power and movement

Next, we split our analysis into trials on which the players approached and trials where they made no movement. Across the brain, we did not find a difference between approach and avoidance trials, neither at trial start nor at token appearance. Because the number of trials is unbalanced for the combinations of approach–avoidance and threat levels, we extracted power from the significant clusters on a trial-by-trial basis, and analyzed these data in a hierarchical linear mixed-effects model. This analysis replicated the impact of threat level (*p* < 0.01 for both clusters) but revealed no effect of approach–avoidance. Next, we included approach latency into a model on trials on which participants approached the token. Approach latency did not relate to power in the mid-hippocampus, but it significantly related to power in the posterior hippocampus (*F*_(1, 6894)_ = 6.5, *p* = 0.01). However, the effect of threat level was still significant in this analysis (*p* < 0.05 in both clusters). Together, this suggests that the threat level–power relation is not better explained by approach–avoidance or by approach latency.

#### Threat level and potential loss

To more directly compare the effects of threat level and token loss, we computed AIC as approximation to the evidence for a model including only threat level, or only potential loss. This analysis was done in the entire anatomical region-of-interest to avoid any bias induced by the cluster-defining contrast (Fig. 3*D*). Across the bilateral hippocampus, threat level explained more variance than potential loss in power averaged across voxels, and in 50% of individual voxels, whereas potential loss explained more variance in 2% of voxels. This suggests theta power in the hippocampus is more closely related to threat level than to token loss, as expected from the initial analysis.

#### Time-frequency decomposition

To decompose the threat level–power relation, we split the beamformer into frequency bands. A threat level × frequency band ANOVA replicated the impact of threat level (*p* < 0.05 for both clusters) and showed that power was unequally distributed across frequency bands, as expected from their definition (*p* < 0.05 for both clusters). Notably, we found a threat-level × frequency interaction (mid-hippocampus: *F*_(8,176)_ = 3.32; *p* = 0.001; posterior hippocampus: *F*_(8,176)_ = 2.29; *p* = 0.024), suggesting that the threat level–power relation was not equally distributed among frequency bands. For the mid-hippocampus cluster, *post hoc t* tests revealed a significant (*p* < 0.05) impact of threat level on power in the beta, gamma, and high-gamma band. For the posterior hippocampus cluster, we found an impact of threat level on the theta and gamma bands.

**Figure 4.**
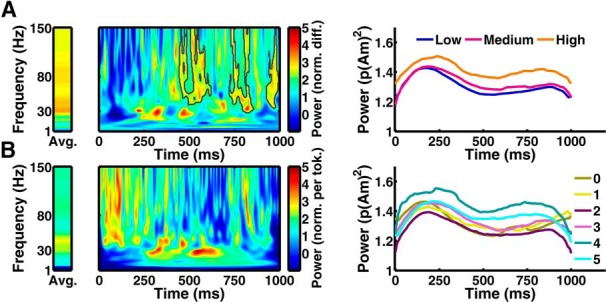
Time frequency decomposition of extracted source activity from mid-hippocampus cluster. ***A***, Power difference (high vs low threat) averaged across time points, and for the entire time-frequency window, shown as condition difference normalized by SE of that difference over participants. Significant clusters from a cluster-level permutation test are outlined in black. Time course of power, averaged over all frequencies, is shown in absolute units for the three threat levels. ***B***, Power increase per additional token, averaged across time points, and for the entire time-frequency window, shown as regression slope normalized by the SE of that regression slope over participants. Time course of power, averaged over all frequencies, is shown in absolute units for the six potential losses.

We then extracted the estimated time course of all sources within the significant clusters, and computed a time-frequency decomposition. Statistical contrasts were corrected for multiple comparisons using a cluster-level permutation test. In the mid-hippocampus cluster, an impact of threat level was particularly pronounced >25 Hz and after ∼450 ms (Fig. 4*A*). Cluster-level tests on averages across time, or frequencies, revealed that the effect of threat level was particularly pronounced at frequencies between 23 and 150 Hz, and for all time points between 297 and 900 ms. For the posterior hippocampus cluster, this analysis revealed no clusters of interest.

### Theta modulation of gamma power

For the remaining analyses, we focused on the mid-hippocampus cluster, which is spatially close to, and shows the same threat–power relation as, the ventral hippocampus subregion for which previous rodent work has revealed oscillatory coupling. Extracted gamma power from this cluster appeared to be modulated by lower frequencies (Fig. 4*A*), reminiscent of a theta phase–gamma power coupling observed in rodent anxiety models (Stujenske et al., 2014). To analyze this modulation, we averaged gamma (30–80 Hz) power for each point in time, and analyzed spectral modulation (1–30 Hz) of this gamma power envelope for each trial. Averaged within frequency bands, this analysis revealed overall stronger modulation of gamma power envelope in theta/alpha than in beta band (main effect frequency: *F*_(2,44)_ = 11.46, *p* < 0.001, *post hoc* test theta > beta band: *t*_(22)_ = 3.65, *p* = 0.001; alpha > beta band: *t*_(22)_ = 3.70, *p* = 0.001). Furthermore, gamma envelope appeared to be particularly more modulated at theta/alpha than at beta frequencies when threat level was high (interaction threat level × frequency: *F*_(2,44)_ = 4.94, *p* = 0.012, *post hoc* test theta > beta band: *t*_(22)_ = 2.29, *p* = 0.032; alpha > beta band: *t*_(22)_ = 2.30, *p* = 0.030). Strikingly, when first averaging gamma envelope within conditions and then doing the spectral decomposition, we found the same pattern of results. This suggests theta/alpha modulation of gamma power is time-locked to token presentation.

#### Theta synchronization between mPFC and hippocampus

**Figure 5.**
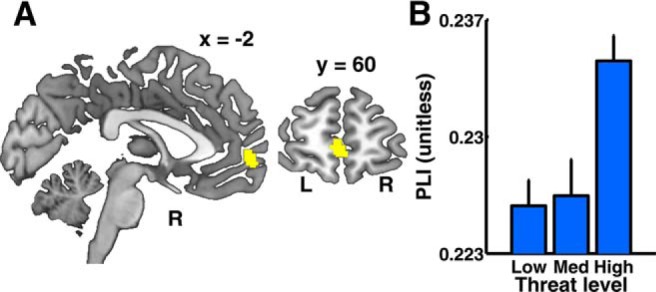
Phase coupling of mid-hippocampus cluster with mPFC. ***A***, At token appearance, PLI between mid-hippocampus and mPFC increases with threat level. Significant cluster (yellow, region BA10) overlayed on a template brain image (*p* < 0.05, cluster level corrected for familywise error in a mPFC mask as defined in the AAL toolbox). ***B***, Mean PLI value in the significant cluster for three different threat levels, corrected for medium threat level. PLI is shown as condition mean/SE of difference from participant mean.

Finally, based on previous findings that hippocampal and mPFC theta oscillations synchronize more strongly when threat is higher, we computed the PLI between the maximum source in the mid-hippocampus cluster, and all other sources in the brain, and analyzed the resulting PLI images. We found an mPFC area for which PLI increased with higher threat level (*p* < 0.05; cluster-level corrected for familywise error within anatomically defined mPFC; Fig. 5*A*,*B*; Table 2). This result appeared to reflect phase coupling and was not driven by differences in mean phase angle between the two conditions. There was no impact of potential loss on PLI.

## Discussion

Neural oscillations in hippocampus, and hippocampus–mPFC theta synchronization is often observed in rodent approach–avoidance conflict tests of anxiety. In the present study, we investigated occurrence and synchronization of hippocampal oscillations in humans to elucidate their possible role in behavioral control during approach–avoidance conflict.

As a first result, we show that hippocampal power is linearly predicted by learned threat probability, but not by explicitly signaled threat magnitude, two prominent aversive features in this approach–avoidance conflict test. The locations of the significant medial temporal lobe (MTL) clusters were consistent with mid-hippocampus (positive relation to threat probability) and posterior tip of hippocampus (negative relation to threat probability). Crucially and extending previous studies, this finding cannot be explained by behavioral responses, i.e., the initiation and latency of virtual movements. Although in the rodent model, theta and gamma oscillations have been linked to aversive and safe features of a situation (Likhtik et al., 2014; Stujenske et al., 2014), our results are more specific and restrict the relevant aversive features to threat probability. Because threat probability is learned in our task, and threat magnitude is explicitly signaled to participants on each trial, this may suggest that a possible function of hippocampus in this task is restricted to situations involving retrieval of threat memories. This result resonates with results from more experiments that more specifically address threat memory, namely fear conditioning. Here, amygdala–mPFC synchronization appears to signal threat memory (Likhtik et al., 2014). It has been further shown that optogenetic inhibition of basolateral amygdala projection terminals in the animals' ventral hippocampus disrupts anxiety-like behaviors, suggesting that hippocampus may be interacting with amygdala to receive threat related memories (Felix-Ortiz et al., 2013). Interestingly, in our dataset, hippocampal responses are only seen when a token appeared to create behavioral conflict, but not at the trial start when all features of the situation were already signaled. This may indicate that the hippocampus is specifically involved in monitoring behavioral conflict, including a retrieval of threat memory (Oehrn et al., 2015; Ito and Lee, 2016).

We can rule out that our results relate to conflict alone because threat magnitude and threat probability both share a relation with conflict, but only probability relates to hippocampal power. Also, by removing most spatial features from the paradigm used in the current study, we are able to firmly rule out that the treat probability–hippocampal power relation is related to an impact of spatial navigation. As a limitation, whereas the location of the significant MTL cluster is consistent with the mid-hippocampus, more precise MEG methods may be required to corroborate the exact location within the MTL. Furthermore, the cluster was not significant in a whole-brain analysis. Although we had strong a priori reasons to focus on the hippocampus as region-of-interest, replication with high-precision MEG (Troebinger et al., 2014a,b) could possibly strengthen this finding.

Interestingly, the threat level–power relationship was most pronounced in the gamma band. Hippocampal gamma oscillations are often coupled to theta phase (Lisman and Jensen, 2013), as also shown with MEG (Poch et al., 2011). However, there is a sparsity of rodent literature on this coupling in approach–avoidance conflict. It appears that rodent amygdala gamma power is coupled to either local or mPFC theta rhythms, depending on threat (Stujenske et al., 2014). However, amygdala gamma power in this previous study showed a negative relation to threat, and may thus be distinct from the gamma power effects we observe here. Crucially, we find stronger theta modulation of hippocampal gamma power when threat is higher, suggesting theta–gamma coupling.

Finally, we identify a positive relation of hippocampal–mPFC coupling with threat level, i.e., hippocampal and mPFC rhythms appear more synchronized when threat is higher. This is in keeping with rodent findings (Adhikari et al., 2010; Padilla-Coreano et al., 2016), which we crucially extend by demonstrating the lack of a relation between potential loss and hippocampus–mPFC coupling. This may indicate that this coupling is intricately linked to a situation in which threat memories are retrieved. A difference in our report from findings using optogenetic manipulations in rodents (Padilla-Coreano et al., 2016) is that we did not assess directionality of this coupling. Interestingly, the location of mPFC coupling with hippocampus in BA10 reflects an area found to synchronize with hippocampus during value-based decision-making (Guitart-Masip et al., 2013).

In a different human approach–avoidance test involving spatial navigation (“stay and play” game), we have previously shown that hippocampal blood oxygenation measured by functional magnetic resonance imaging relates to threat level (Bach et al., 2014), just like hippocampal oscillatory power in the current study. The power effect in the current study was broadly limited to gamma band (24–150 Hz), and oscillations in this frequency range show a robust relationship with BOLD responses (Boorman et al., 2015; Hutchison et al., 2015; Scheeringa et al., 2016), rendering the two findings rather consistent. Interestingly, other fMRI studies on anxiety-like behavior in approach–avoidance conflict have also suggested an encoding of conflict per se and/or action tendencies in multivariate patterns of hippocampal BOLD signal (O'Neil et al., 2015; Loh et al., 2017). Control analyses of our data revealed that such features were not represented in hippocampal power, or hippocampal–mPFC coupling in our task. Interestingly, both of these latter studies addressed a slightly different situation, in which a decision is being abstractly communicated to the computer via button press, whereas the motor execution of that button press has no impact on outcome. Our initial stay and play task (Bach et al., 2014), as in most rodent anxiety tests, required specific motor behaviors. Although largely removing the element of spatial navigation in our current task, motor execution is still crucial and has a major impact on outcomes: if players move later they are less likely to obtain the token; if the return to the safe place later they are more likely to get caught. This task demand may rely on partly distinct neural control than the more abstract demands in (O'Neil et al., 2015; Loh et al., 2017), which share some analogy with specific operant rodent tests (Geller and Seifter, 1960; Vogel et al., 1971). Indeed, using similar operant conflict tests, a more recent rodent and (human and nonhuman) primate literature has not implicated the hippocampus in approach–avoidance decision-making at all, and rather highlighted contributions of anterior cingulate, and of striosomes in the basal ganglia (Amemori and Graybiel, 2012; Amemori et al., 2015; Aupperle et al., 2015; Friedman et al., 2015). However, as a limitation to this distinction, a role of the anterior cingulate rather than hippocampus has also been highlighted in an approach–avoidance task with naturalistic continuous responses (Gonen et al., 2016). Reconciling spatial, mnemonic, conflict processing, and behavioral control functions of the hippocampus may therefore require more elaborated experimental scenarios (Ito and Lee, 2016).

To summarize, we used a virtual computer game simulating biologically relevant approach–avoidance conflict in humans, to investigate functional role of hippocampal oscillations. We show that hippocampal power linearly relates to learned threat probability in a location consistent with the right mid-hippocampus. This is not paralleled by threat magnitude and cannot be explained by virtual movement, action tendencies, or conflict per se. This result mainly appears to stem from the gamma band, which shows stronger theta modulation when threat is higher. Finally, theta oscillations in this location are more synchronized with mPFC at higher threat probability. Thus it appears that the role of hippocampal oscillations and their synchronization with mPFC in approach–avoidance situations is restricted to the retrieval of threat memory. This resonates with recent attempts to elucidate the role of brain synchronization in defensive behavior.
